# Chronic Disease Self-Management Program in the Workplace: Opportunities for Health Improvement

**DOI:** 10.3389/fpubh.2014.00179

**Published:** 2015-04-27

**Authors:** Matthew Lee Smith, Mark G. Wilson, David M. DeJoy, Heather Padilla, Heather Zuercher, Phaedra Corso, Robert Vandenberg, Kate Lorig, Marcia G. Ory

**Affiliations:** ^1^Workplace Health Group, Department of Health Promotion and Behavior, College of Public Health, The University of Georgia, Athens, GA, USA; ^2^Department of Health Policy and Management, College of Public Health, The University of Georgia, Athens, GA, USA; ^3^Department of Management, Terry College of Business, The University of Georgia, Athens, GA, USA; ^4^Stanford Patient Education Research Center, Department of Medicine, Stanford School of Medicine, Palo Alto, CA, USA; ^5^Department of Health Promotion and Community Health Sciences, Texas A&M Health Science Center School of Public Health, College Station, TX, USA

**Keywords:** chronic disease self-management, evidence-based program, workplace wellness, evaluation, translational research

## Abstract

Disease management is becoming increasingly important in workplace health promotion given the aging workforce, rising chronic disease prevalence, and needs to maintain a productive and competitive American workforce. Despite the widespread availability of the Chronic Disease Self-Management Program (CDSMP), and its known health-related benefits, program adoption remains low in workplace settings. The primary purpose of this study is to compare personal and delivery characteristics of adults who attended CDSMP in the workplace relative to other settings (e.g., senior centers, healthcare organizations, residential facilities). This study also contrasts characteristics of CDSMP workplace participants to those of the greater United States workforce and provides recommendations for translating CDSMP for use in workplace settings. Data were analyzed from 25,664 adults collected during a national dissemination of CDSMP. Only states and territories that conducted workshops in workplace settings were included in analyses (*n* = 13 states and Puerto Rico). Chi-squared tests and *t*-tests were used to compare CDSMP participant characteristics by delivery site type. CDSMP workplace participant characteristics were then compared to reports from the United States Bureau of Labor Statistics. Of the 25,664 CDSMP participants in this study, 1.7% (*n* = 435) participated in workshops hosted in worksite settings. Compared to CDSMP participants in non-workplace settings, workplace setting participants were significantly younger and had fewer chronic conditions. Differences were also observed based on chronic disease types. On average, CDSMP workshops in workplace settings had smaller class sizes and workplace setting participants attended more workshop sessions. CDSMP participants in workplace settings were substantially older and a larger proportion were female than the general United States workforce. Findings indicate opportunities to translate CDSMP for use in the workplace to reach new target audiences.

## Introduction

Chronic diseases are multi-dimensional and affect all aspects of people’s lives, especially work (1, 2). People with chronic diseases are constantly required to make decisions that affect their health, which have ramifications for work performance and employability. It has been reported that, depending on the chronic condition involved, between 22 and 49% of employees experience difficulties meeting physical work demands, while between 27 and 58% have problems meeting psychosocial work requirements (3). These problems can lead to job loss or premature departure from the workforce.

To complicate matters, the American workforce is aging. The Bureau of Labor Statistics (BLS) predicts that between 2006 and 2016, the number of workers aged 65–74 years will increase by 83%, and those aged 75 and older will increase by 84% (4). Many of these older workers will have one or more chronic diseases; 77% of older adults currently have two or more comorbidities (5). Recognizing the growing prevalence of obesity and other cardiovascular risk factors (e.g., hypertension, hypercholesterolemia, diabetes) and related chronic conditions among working-aged Americans (6), interventions are needed to arm middle-aged and older employees with skills and strategies to manage their diseases and associated symptoms.

Disease management is increasingly recognized as an important component of workplace health promotion given our aging workforce, the prevalence of chronic conditions, and the importance of maintaining a productive and competitive American workforce (7–11). Currently, most workplace-based disease management programs are offered by health insurance providers and operate largely independent of other on-site health promotion activities (2). Mounting evidence supports the effectiveness and growing importance of disease management programing in workplace settings (12–15). Unfortunately, workplace-delivered disease management activities may have limited reach because they are expensive and require medically trained providers/facilitators (2). These activities may also have narrow scopes (i.e., focus on one specific disease or condition).

Stanford’s Chronic Disease Self-Management Program (CDSMP) is among the most widely disseminated and researched evidence-based programs (16, 17) and is extremely effective in helping individuals better manage their chronic disease and related complications (18–20). Developed based on over 20 years of research at Stanford University, CDSMP is currently offered in over 30 countries and a variety of languages^1^. Traditionally delivered through the aging services network, this robust program has been delivered in a wide variety of community settings (e.g., senior centers, healthcare organizations, residential facilities, faith-based organizations, and tribal centers). CDSMP has the advantages of being inexpensive and easily disseminated. It is not disease or condition specific and can be delivered effectively by lay leaders with minimal training using a train-the-trainer model.

To date, CDSMP has not been widely implemented by workplace settings or incorporated into workplace health promotion programing (21). In part, low-implementation rates in workplace settings may be a consequence of CDSMP being primarily delivered through the aging services network, which predominately targets older adults, many of whom may no longer in the workforce. Additionally, the standard CDSMP structure and format (i.e., 2.5 h sessions, once a week for six consecutive weeks) may not appear amenable to widespread implementation in work organizations. For these reasons, it is important to investigate characteristics of CDSMP uptake in workplace settings and explore opportunities for reaching and better serving the American workforce.

Using data from the first 100,000 participants collected during a 2-year national dissemination of CDSMP, the primary purpose of this study was to compare personal and delivery characteristics of adults who attended CDSMP in the workplace relative to other settings (e.g., senior centers, healthcare organizations, residential facilities). To contextualize CDSMP implementation in workplace settings, this study also contrasts characteristics of CDSMP workplace participants relative to those of the greater United States workforce. Building upon these findings, we highlight potential opportunities for translating CDSMP for use in workplace settings to overcome traditional barriers, reach new customer markets, and improve work performance indicators while maintaining the program’s well-documented effectiveness.

## Materials and Methods

### Program description

The CDSMP has been introduced and disseminated in the United States as a method to empower patients with self-management skills to deal with their chronic conditions (22). Drawing upon social learning theory (23), CDSMP is an evidence-based, peer-led intervention consisting of six highly participative classes held for 2.5 h each, once a week, for six consecutive weeks (22). CDSMP has resulted in improved healthcare and health (18, 20), while potentially saving healthcare costs (19).

### Data source and study population

Cross-sectional data for this study were obtained from a nationwide delivery of CDSMP as part of the American Recovery and Reinvestment Act of 2009 (i.e., Recovery Act) *Communities Putting Prevention to Work: Chronic Disease Self-Management Program* initiative (16). The United States Administration on Aging led this initiative in collaboration with the Centers for Disease Control and Prevention (CDC) and the Centers for Medicare and Medicaid Services to support the delivery of CDSMP in 45 states, Puerto Rico, and the District of Columbia (17). This initiative was originally designed to have 50,000 Americans complete at least 4 out of 6 CDSMP sessions between 2010 and 2012 and to embed CDSMP delivery structures into statewide systems (16).

For this study, data were analyzed from 25,664 participants who attended CDSMP workshops in 13 states and 1 territory that delivered the program in workplace settings (i.e., to reduce threats for systematic bias associated with state-specific delivery infrastructures or preferences) and had no missing data for variables of interest. We also utilized 2012 BLS data from the United States Department of Labor to compare CDSMP participant characteristics to those of the larger American workforce^2^.

### Measures

The primary variable of interest in this study was whether or not CDSMP participants attended program workshops in workplace settings. Data from states that did not deliver one or more CDSMP workshops were omitted from study analyses. Among included states, workshop delivery site type was dichotomized into worksite settings and non-worksite settings. Non-worksite settings included senior centers, area agencies on aging (AAA), healthcare organizations, residential facilities, community or multipurpose centers, faith-based organizations, educational institutions, and tribal centers. Other workshop-level variables of interest included the number of participants enrolled in the workshop (i.e., continuous number ranging from 1 to 20 individuals) and the number of workshop sessions attended (i.e., “successful completion” is defined as attending 4 or more of the 6 possible sessions) (20).

Participant characteristics of interest in this study included age (i.e., measured continuously in years as well as categories consistent with those reported by the United States Department of Labor), sex, ethnicity, and race. Rural–urban commuting area codes based on participants’ ZIP were used to categorize participants’ residence (metro vs. non-metro). The number and type of self-reported chronic conditions was also recorded (i.e., arthritis, cancer, depression, diabetes, heart disease, hypertension, lung disease, stroke, osteoporosis, and other chronic conditions).

### Analyses

To compare the characteristics of the participants who attended CDSMP workshops in workplace settings and those who participated in other settings, we used chi-square tests for categorical variables and independent-sample *t*-tests for continuous variables. Only data for the following states and territories were included in analyses: Alabama, Arizona, Florida, Hawaii, Maine, Maryland, Massachusetts, Missouri, New Jersey, North Carolina, Oregon, Puerto Rico, Virginia, and Wisconsin. Statistical analyses for this descriptive study were performed using SPSS (version 21).

## Results

### CDSMP participation in workplace settings

Of the 25,664 participants who attended CDSMP workshops in these 13 states and 1 territory, 435 (1.70%) did so at a workplace setting. As seen in Table 1, the average age of workplace setting participants was 61.12 (±14.69) years. The majority of workplace setting participants was under age 65 years (58.6%), female (80.9%), non-Hispanic (96.1%), and white (61.6%). Over 63% reported living in metro areas. On average, workplace setting participants self-reported having 2.29 (±1.50) chronic conditions, with 41.4% reporting 3 or more coexisting conditions. The most frequently reported chronic conditions were hypertension (43.9%), arthritis (40.0%), diabetes (28.0%), and depression (22.5%). Almost 35% of participants reported some other chronic condition. On average, CDSMP workshop held in workplace settings had 10.82 (±3.89) participants. On average, these participants attended 4.84 (±1.46) of the 6 workshop sessions, with 85.1% successfully completing the workshop.

**Table 1:** **National sample characteristics by CDSMP delivery site type**.

	U.S. Workforce	Total CDSMP	Worksite setting	Non-worksite	χ^2^ or *t*	*P*
	Statistics (2012)^a^	participants	(*n* = 435)	setting (*n* = 25,229)		
		(*n* = 25,664)				
Age	–	67.70 (±14.35)	61.12 (±14.69)	67.82 (±14.31)	9.67	<0.001
Under 50	66.8%	11.2%	20.0%	11.0%	108.82	<0.001
50–64	28.0%	23.9%	38.6%	23.7%		
6574	4.2%	30.2%	20.0%	30.4%		
75+	1.0%	34.7%	21.4%	35.0%		
Sex
Male	53.0%	21.2%	19.1%	21.2%	1.14	0.286
Female	47.0%	78.8%	80.9%	78.8%		
Hispanic ethnicity
No	–	89.0%	96.1%	88.9%	22.70	<0.001
Yes	15.4%	11.0%	3.9%	11.1%		
Race
White	80.5%	66.7%	61.6%	66.8%	85.66	<0.001
African American	11.2%	20.9%	14.0%	21.0%		
Asian/Pacific Islander	5.5%	4.7%	12.9%	4.5%		
American Indian/Alaska naive	–	1.0%	2.1%	0.9%		
Other multiple races	–	6.8%	9.4%	6.7%		
Number of chronic conditions	_	2.50 (±1.65)	2.29 (±1.50)	2.50 (±1.65)	2.90	0.004
0	–	9.2%	9.2%	9.2%	3.94	0.268
1	–	21.4%	24.6%	21.3%		
2	–	23.9%	24.8%	23.9%		
3+	–	45.5%	41.4%	45.6%		
Participant residence					
Metro	–	78.5%	63.4%	78.7%	59.01	<0.001
Non-metro	–	21.5%	36.6%	21.3%		
Number of participant enrolled in workshop	–	12.59 (±4.03)	10.82 (±3.89)	12.62 (±4.03)	9.23	<0.001
Number of sessions attended	–	4.53 (±1.63)	4.84 (±1.46)	4.53 (±1.63)	−4.48	<0.001
Successful completion: no	–	21.8%	14.9%	21.8%	12.16	<0.001
Successful completion: yes	–	78.2%	85.1%	78.1%		
Disease prevalence					
Arthritis	–	47.1%	40.0%	47.2%	8.99	0.003
Cancer	–	10.9%	12.0%	10.9%	0.49	0.486
Depression	–	23.1%	22.5%	23.1%	0.09	0.770
Diabetes	–	32.1%	28.0%	32.1%	3.29	0.070
Heart disease	–	18.6%	16.6%	18.6%	1.18	0.277
Hypertension	–	48.7%	43.9%	48.8%	4.12	0.042
Lung disease	–	19.1%	16.1%	19.1%	2.51	0.113
Stroke	–	5.4%	3.2%	5.4%	4.12	0.042
Osteoporosis	–	14.6%	12.2%	14.7%	2.10	0.148
Other	–	30.3%	34.7%	30.2%	4.07	0.044

*^a^Unadjusted estimates of employed persons (U.S. Bureau of Labor Statistics; http://www.bls.gov/data/#employment)*.

*–, Workforce data not available for comparison purposes*.

Compared to CDSMP participants in non-workplace settings, workplace setting participants were significantly younger and had fewer chronic conditions. A significantly smaller proportion of workplace setting participants had arthritis, hypertension, stroke, whereas a significantly larger proportion of these participants had other chronic condition types. A significantly larger proportion of participants in workplace settings were non-Hispanic and non-white, although a significantly larger proportion of non-workplace setting participants were African American. On average, CDSMP workshops in workplace settings had significantly fewer participants, and participants in workplace settings attended significantly more workshop sessions.

Compared to 2012 estimates from the United States Department of Labor, a larger proportion of CDSMP participants in workplace settings were over age 50 years, female, non-Hispanic, and non-white.

## Discussion

Findings from this descriptive study indicate that CDSMP adoption is low in the workplace, with merely 1.7% of participants in this sample attending workshops in workplace settings. Over 66% of workplace setting participants had two or more chronic conditions, which indicates the need for a widely available, high-quality disease self-management intervention. While significantly smaller proportions of workplace setting participants had arthritis, hypertension, and stroke relative to non-workplace setting participants (conditions more prevalent in the older adult population), it is interesting that rates of self-reported chronic conditions among workplace setting participants were generally comparable to rates among non-workplace setting participants. This aligns with previous reports indicating the American workforce is developing chronic conditions and accruing more comorbidities during their extended time on the job before their delayed retirement (24).

Compared to non-workplace setting participants in this study, workplace setting participants were significantly younger (i.e., on average 6.7 years younger); however, they were substantially older than the American workforce (i.e., 5.2% of the 2012 workforce aged 65 and older compared to 51.4% of CDSMP workplace setting participants). Further, when compared to the American workforce, males and Hispanics were underrepresented in CDSMP workplace settings. This finding highlights potential opportunities to expand program reach to new target audiences. When CDSMP was delivered in workplace settings, a significantly larger proportion of participants successfully completed the workshop relative to those in non-workplace settings (i.e., 85.1% compared to 78.1%). Clearly there is potential to expand CDSMP reach and adoption among the American workforce.

### Opportunities to translate CDSMP for use in the workplace

Agencies such as the CDC are promoting coordinated approaches to workplace health that encompass interventions to address the multi-factorial influences of health risk and employee wellness (25). Aligned with CDC’s goal of increasing the number of science-based initiatives in worksites (26), implementing and evaluating CDSMP in workplace settings is a viable strategy to improve employee health using a proven evidence-based intervention. Even though there is considerable need in worksite health promotion for efficacious disease management programs, CDSMP has not been tested in a format conducive for broad-based worksite dissemination. If CDSMP were appropriately tailored to the needs of middle-aged and older workers and delivered through workplace settings, this translated version would have potential to reduce healthcare utilization and boost work productivity and retention. This combination of benefits coupled with relatively low-delivery costs and scalability should be attractive to almost all employers and employer groups (i.e., leverage for making “a business case” to adopt CDSMP). Further, this model has potential to be extremely cost-effective and yield substantial returns on investment.

### Unique nature of worksites

Although there is considerable potential for offering CDSMP in workplaces, in order to maximize program effectiveness, it needs to be translated to accommodate the unique nature of worksite settings. Generally speaking, the typical worker is paid a certain amount of money, to work a defined period of time, to accomplish specific tasks or outcomes that will benefit the organization’s goals and enable them to support themselves and their families. Organizations, in turn, are focused on maximizing the outcomes and minimizing the costs to achieve those outcomes, most of which are driven by the people, environment, and materials needed to produce the product or outcome. So, both the worker and organization have a strong economic incentive, time constraints, and interrelated goals and/or outcomes that are restricted by the environment in which they operate. These factors vary from organization to organization and job to job. As a result, any intervention implemented in worksites must be tailored to these unique characteristics. For CDSMP to be effective in worksite settings, it must be cost-effective, not too disruptive of work schedules, and achieve varying work-related outcomes (both individual and organizational). And, most importantly, it must do so within the constraints of the workplace environment.

### CDSMP translation

As with any translation, it is imperative to maintain the program’s integrity, which assures that the original intervention effects will be achieved. This could be accomplished by keeping the content, program duration, and number of contact hours constant, but modifying the session length (and thereby increasing the number of sessions) and incorporating worksite-specific strategies. This translation should also include efforts to complement existing workshop content to include topics, skills, and examples more relevant to working-aged individuals. It would afford researchers and evaluators an opportunity to introduce and assess new outcomes measures related to work performance and productivity. Such modifications may overcome barriers to workplace adoption as well as foster more universal cross-industry appeal in small rural jobsites and Fortune 500 companies alike. In the event that a workplace-based CDSMP were created, the new intervention would need to be standardized (as with any evidence-based program), with careful attention given to implementation manuals, fidelity standards, leader training (i.e., new and/or “bridge” trainings for T-trainers, master trainers, lay leaders), workshop materials, and evaluation tools and protocol.

#### Translation benefits

A key advantage of offering CDSMP to working adults would be that the program could reach younger individuals and those who are earlier in the time course of their chronic conditions, thereby reducing the likelihood of costly and debilitating complications. A workplace-based CDSMP has the potential to reach large numbers of working adults, which is not occurring through current workplace disease management delivery models or traditional CDSMP delivery channels.

Although delivered in workplace settings, a workplace-based CDSMP would benefit from ongoing collaboration with the aging services network and local community-based organizations to ensure long-term program sustainability. Further, a workplace-based CDSMP would readily complement other existing on-site health promotion initiatives that target healthy lifestyle behaviors and healthy decision-making.

### Limitations

As with any study, there were limitations that should be addressed. Because of the grand scale nature of this national initiative, only self-report sociodemographics and administrative records were collected. No outcome data were obtained. Of the 45 states and 2 territories involved in this study, only 13 states and Puerto Rico offered workshops in workplace settings, thus, only data from these areas were included in study analyses to reduce possible bias when making comparisons. However, because 2012 BLS data contained data from all states and territories, comparisons with CDSMP participant data were less than ideal. Further, BLS data only contained a few variables to which CDSMP participant data could be compared (e.g., no information about rurality or disease diagnoses). This limited these authors’ ability to fully realize the aims of this study.

## Conclusion

This study provides a unique glimpse into the under-explored realm of CDSMP delivered in workplace settings. Findings suggest considerable opportunities for translating CDSMP for use in workplace settings to overcome traditional barriers, reach new target audiences, and improve work performance indicators while maintaining the program’s effectiveness. While the recommendations put forth in this paper are those of the authors, additional workplace-based CDSMP translation efforts are inevitable.

## Conflict of Interest Statement

The authors declare that the research was conducted in the absence of any commercial or financial relationships that could be construed as a potential conflict of interest.

This paper is included in the Research Topic, “Evidence-Based Programming for Older Adults.” This Research Topic received partial funding from multiple government and private organizations/agencies; however, the views, findings, and conclusions in these articles are those of the authors and do not necessarily represent the official position of these organizations/agencies. All papers published in the Research Topic received peer review from members of the Frontiers in Public Health (Public Health Education and Promotion section) panel of Review Editors. Because this Research Topic represents work closely associated with a nationwide evidence-based movement in the US, many of the authors and/or Review Editors may have worked together previously in some fashion. Review Editors were purposively selected based on their expertise with evaluation and/or evidence-based programming for older adults. Review Editors were independent of named authors on any given article published in this volume.

## Footnotes

^1^http://patienteducation.stanford.edu/programs/cdsmp.html

^2^http://www.bls.gov/data/#employment

## References

[1] Detaille SI van der Gulden JWJ Engels JA Heerkens YF van Dijk FJ. Using intervention mapping (IM) to develop a self-management programme for employees with a chronic disease in the Netherlands. BMC Public Health (2010) 10:353. 10.1186/1471-2458-10-353 20565925 PMC2908090

[2] Richling DE Disease management for employed populations. 2nd ed In: Pronk NP, editor. ACSM’s Worksite Health Handbook: A Guide to Building Healthy and Productive Companies. Champaign, IL: Human Kinetics (2009). p. 286–95.

[3] Lerner D Allaire S Reisine S. Work disability resulting from chronic health conditions. J Occup Environ Med (2005) 47(3):253–64. 10.1097/01.jom.0000150206.04540.e7 15761321

[4] Bureau of Labor Statistics. Older Workers [Internet]. (2008). Available from: http://www.bls.gov/spotlight/2008/older_workers/

[5] Agency for Health Research and Quality. Medical Expenditure Panel Survey Household Component: 2010 Full Year Consolidated Data File. Rockville, MD (2010). Available from: http://meps.ahrq.gov/data_stats/download_data_files_detail.jsp?cboPufNumber=HC-138

[6] Bhattacharya J Choudhry K Lakdawalla D Chronic disease and severe disability among working-age populations. Med Care (2008) 46(1):92–100 10.1097/MLR.0b013e3181484335 18162861

[7] Goetzel RZ Pei X Tabrizi MJ Henke RM Kowlessar N Nelson CF Ten modifiable health risk factors are linked to more than one-fifth of employer-employee health care spending. Health Aff (2012) 31(11):2474–84. 10.1377/hlthaff.2011.0819 23129678

[8] Special Committee on Health, Productivity, and Disability Management. Healthy workforce/healthy economy: The role of health, productivity, and disability management in addressing the nation’s health care crisis. J Occup Environ Med (2009) 51(1):114–9 10.1097/JOM.0b013e318195dad2 19136880

[9] Collins JJ Baase CM Sharda CE Ozminkowski RJ Nicholson S Billotti GM The assessment of chronic health conditions on work performance, absence, and total economic impact for employers. J Occup Environ Med (2005) 47(6):547–57. 10.1097/01.jom.0000166864.58664.29 15951714

[10] Hoffman C Rice D Sung H-Y Persons with chronic conditions. Their prevalence and costs. J Am Med Assoc (1996) 276(18):1473–9 10.1001/jama.276.18.1473 8903258

[11] Hwang W Weller W Ireys H Anderson G. Out-of-pocket medical spending for care of chronic conditions. Health Aff (2001) 20(6):267–78. 10.1377/hlthaff.20.6.267 11816667

[12] Nyman JA Abraham JM Jeffery MM Barleen NA. The effectiveness of a health promotion program after 3 years: evidence from the University of Minnesota. Med Care (2012) 50(9):772–8. 10.1097/MLR.0b013e31825a8b1f 22683588

[13] Caloyeras JP Liu H Exum E Broderick M Mattke S. Managing manifest diseases, but not health risks, saved PepsiCo money over seven years. Health Aff (2014) 33(1):124–31. 10.1377/hlthaff.2013.0625 24395944

[14] Varekamp I Verbeek JH van Dijk FJH. How can we help employees with chronic diseases to stay at work? A review of interventions aimed at job retention and based on an empowerment perspective. Int Arch Occup Environ Health (2006) 80(2):87–97. 10.1007/s00420-006-0112-9 16758194

[15] Pelletier K A review and analysis of the clinical and cost-effectiveness studies of comprehensive health promotion and disease management programs at the worksite: update VI 2000-2004. J Occup Environ Med (2005) 47(10):1051–8 10.1097/01.jom.0000174303.85442.bf 16217246

[16] Ory MG Smith ML Patton K Lorig K Zenker W Whitelaw N Self-management at the tipping point: Reaching 100,000 Americans with evidence-based programs. J Am Geriatr Soc (2013) 61(5):821–3 10.1111/jgs.12239 23672545

[17] ARRA. Communities Putting Prevention to Work: Chronic Disease Self-Management Program [Internet]. US Department of Health and Human Services, Administration on Aging (2012). Available from: https://www.cfda.gov/index?s=program&mode=form&tab=core&id=04e3dca0a3218583cc472c28f5d26644

[18] Ory M Ahn S Jiang L Lorig K Ritter P Laurent D National study of chronic disease self-management. J Aging Health (2013) 25(7):1258–74 10.1177/0898264313502531 24029414

[19] Ahn S Basu R Smith ML Jiang L Lorig K Whitelaw N The impact of chronic disease self-management programs: healthcare savings through a community-based intervention. BMC Public Health (2013) 13:1141. 10.1186/1471-2458-13-1141 24314032 PMC3878965

[20] Ory MG Ahn S Jiang L Smith ML Ritter PL Whitelaw N Successes of a national study of the chronic disease self-management program: meeting the triple aim of health care reform. Med Care (2013) 51(11):992–8. 10.1097/MLR.0b013e3182a95dd1 24113813

[21] Smith ML Ory MG Ahn S Kulinski KP Jiang L Horel S National dissemination of chronic disease self-management education programs: an incremental examination of delivery characteristics. Front Public Health (2015) 2:227 10.3389/fpubh.2014.00227. PMC4410345 25964923

[22] Lorig K Holman H Sobel D Laurent D Living a Healthy Life with Chronic Conditions. 3rd ed Boulder, CO: Bull Publishing Company (2006).

[23] Bandura A Social cognitive theory of self-regulation. Organ Behav Hum Decis Process (1991) 50(2):248–87 10.1016/0749-5978(91)90022-L

[24] Sliverstein M Meeting the challenges of an aging workforce. Am J Ind Med (2008) 51:269–80 10.1002/ajim.20569 18271000

[25] Centers for Disease Control and Prevention [Internet]. *Workplace Health Model* (2013). Available from: http://www.cdc.gov/workplacehealthpromotion/model/index.html

[26] Centers for Disease Control and Prevention [Internet]. *About Work@Health* (2014). Available from: http://www.cdc.gov/workathealth/about.html

